# *In vitro* binding of *Sorghum bicolor* transcription factors ABI4 and ABI5 to a conserved region of a *GA 2-OXIDASE* promoter: possible role of this interaction in the expression of seed dormancy

**DOI:** 10.1093/jxb/ert347

**Published:** 2013-10-22

**Authors:** Renata Cantoro, Carlos Daniel Crocco, Roberto Luis Benech-Arnold, María Verónica Rodríguez

**Affiliations:** ^1^IFEVA, Facultad de Agronomía, Universidad de Buenos Aires, CONICET, Av. San Martín 4453 (C1417DSE) CABA, Argentina; ^2^Cátedra de Cultivos Industriales, Facultad de Agronomía, Universidad de Buenos Aires, Av. San Martín 4453 (C1417DSE) CABA, Argentina

**Keywords:** ABI4, ABI5, abscisic acid, *GA 2-oxidase*, germination, gibberellins, seed dormancy, *Sorghum bicolor*.

## Abstract

The precise adjustment of the timing of dormancy release according to final grain usage is still a challenge for many cereal crops. Grain sorghum [*Sorghum bicolor* (L.) Moench] shows wide intraspecific variability in dormancy level and susceptibility to pre-harvest sprouting (PHS). Both embryo sensitivity to abscisic acid (ABA) and gibberellin (GA) metabolism play an important role in the expression of dormancy of the developing sorghum grain. In previous works, it was shown that, simultaneously with a greater embryo sensitivity to ABA and higher expression of *SbABA-INSENSITIVE 4* (*SbABI4*) and *SbABA-INSENSITIVE 5* (*SbABI5*), dormant grains accumulate less active GA_4_ due to a more active GA catabolism. In this work, it is demonstrated that the ABA signalling components SbABI4 and SbABI5 interact *in vitro* with a fragment of the *SbGA 2-OXIDASE 3* (*SbGA2ox3*) promoter containing an ABA-responsive complex (ABRC). Both transcription factors were able to bind the promoter, although not simultaneously, suggesting that they might compete for the same *cis*-acting regulatory sequences. A biological role for these interactions in the expression of dormancy of sorghum grains is proposed: either SbABI4 and/or SbABI5 activate transcription of the *SbGA2ox3* gene *in vivo* and promote SbGA2ox3 protein accumulation; this would result in active degradation of GA_4_, thus preventing germination of dormant grains. A comparative analysis of the 5′-regulatory region of GA2oxs from both monocots and dicots is also presented; conservation of the ABRC in closely related GA2oxs from *Brachypodium distachyon* and rice suggest that these species might share the same regulatory mechanism as proposed for grain sorghum.

## Introduction

Seed dormancy is considered as the failure of an intact viable seed to complete germination under conditions that are otherwise favourable for non-dormant seeds (Bewley, 1997). In wild plants from temperate habitats, seed dormancy improves their fitness as it avoids the occurrence of germination under unfavourable conditions, reduces intraspecific competition, and gives species the opportunity to survive natural catastrophes. On the other hand, the process of domestication has pushed towards a fast and uniform germination, followed by rapid seedling establishment and high crop yields (Finkelstein *et al.*, 2008). This has been achieved through the selection of genotypes with a briefer dormancy. However, low dormancy levels before grain harvest can also lead to pre-harvest sprouting (PHS) if adequate temperature and humid conditions prevail during late maturation in the field. For these reasons, the precise adjustment of the timing of dormancy release according to grain usage requirements appears to be a trait of great importance for crop production.

It is widely documented that abscisic acid (ABA) and gibberellins (GAs) play an antagonistic role in the control of seed germination in species that display physiological dormancy (reviewed in Finch-Savage and Leubner-Metzger, 2006; Finkelstein *et al.*, 2008; Nambara *et al.*, 2010). Current knowledge on how GA and ABA control seed germination highlights the importance of inactivation of germination repressors for germination to take place. During dormancy release, ABA levels or sensitivity decline, while an enhanced response to GAs takes place (reviewed by Finch-Savage and Leubner-Metzger 2006). Moreover, it is precisely the ABA–GA balance which determines the expression of dormancy during seed imbibition in many species, including cereals (reviewed by Finch-Savage and Leubner-Metzger, 2006; Finkelstein *et al.*, 2008; Nambara *et al.*, 2010).

Grain sorghum [*Sorghum bicolor* (L.) Moench], like many other cereals, shows wide intraspecific variability in the pattern of dormancy release and PHS response, some genotypes being very susceptible and others resistant to PHS. Sorghum caryopses display coat-imposed physiological dormancy, and removal of the pericarp and endosperm leads to rapid embryo germination (Steinbach *et al.*, 1995). Previous results have shown that embryonic ABA levels measured during grain incubation are not related to their dormancy level (Gualano *et al.*, 2007; Rodríguez *et al.*, 2009). In contrast, changes in embryo sensitivity to ABA correlated with the pattern of dormancy release of intact grains throughout development, for both sprouting-resistant and sprouting-susceptible genotypes (Steinbach *et al.*, 1995; Gualano *et al.*, 2007; Rodríguez *et al.*, 2009). In agreement with these results, Rodríguez *et al.* (2009) reported that the transcription of the sorghum genes *ABI3/VP1*, *ABI4*, *ABI5*, and *PKABA1* (positive regulators of ABA signalling) is stimulated during incubation of dormant grains of the sprouting-resistant inbred line (IS9530), but not in grains of the less dormant, sprouting-susceptible line RedlandB2. In particular, these authors pointed out that the expression of *SbABA-INSENSITIVE 4* (*SbABI4*) and *SbABI5* was transiently induced in IS9530 caryopses after 2–3 d of incubation, together with a similar increase in SbABI5 protein levels.

Genetic analysis of *Arabidopsis* aba-insensitive (*abi*) mutants revealed that the transcription factors ABI4 and ABI5 do not have an effect on dormancy induction or release but have a fundamental role in ABA-mediated inhibition of germination and seed maturation (Brocard-Gifford *et al.*, 2003; Penfield *et al.*, 2006; Piskurewicz *et al.*, 2008; Daszkowska-Golec and Szareijo, 2013). ABI5 is a basic leucine zipper (bZIP) transcription factor, known to be involved in regulating germination in response to ABA and stress (Finkelstein and Rock, 2002). The leucine zipper domain of ABI5 is involved in protein dimerization, which is necessary for DNA binding to occur. Like other bZIP proteins, ABI5 binds ACGT core sequence elements, called ABA response elements or ABREs (Guiltinan *et al.*, 1990; Mundy *et al.*, 1990), found in many ABA-responsive promoters (reviewed by Shinozaki *et al.*, 2007). On the other hand, ABI4 is a member of the APETALA2 domain family and it has been reported that the *Arabidopsis abi4* mutant showed altered expression of ABA-responsive genes such as *Em6* (Finkelstein, 1994). *In vitro* analysis of maize ABI4 binding sites revealed a CACCG consensus sequence, named coupling element 1 (CE1; Niu *et al.*, 2002), and it has also been demonstrated that ABI4 binds to a sequence related to the S-box (CACYKSCA) (Bossi *et al.*, 2009). However, Reeves *et al.* (2011) recently found that the promoters of many ABI4-regulated genes lacked these previously identified ABI4 binding sites, but were enriched for ABREs (bZIP binding sites), and they identified a group of genes that are synergistically co-regulated by ABI4 and bZIP proteins. Hence, it has been suggested that the minimal ABRC (ABA response complex) found in many promoters induced by ABA (such as *HVA1* and *HVA22*) is composed of an ACGT core element (ABRE) and a coupling element (Shen and Ho, 1995; Shen *et al.*, 1996). Similar ABRC configurations were found in the promoters of other ABA response genes such as *Em* genes (Guiltinan *et al.*, 1990), *Rab17* (Busk *et al.*, 1997), and *Rab28* (Busk and Pagés, 1998). Other elements that have been identified in ABA-inducible promoters include RY/Sph, MYC, and MYB elements (reviewed in Cutler *et al.*, 2010). In particular, RY/Sph repeat elements have been found to be crucial for seed-specific promoters (Baumlein *et al.*, 1992; Ezcurra *et al.*, 1999).

As mentioned above, not only ABA but also GAs have a role in dormancy expression in the imbibed seed. In grain sorghum, Benech-Arnold *et al.* (2000) reported that embryo sensitivity to GA_3_ (measured as the ability of increasing GA_3_ concentrations to revert inhibition of embryo germination by 50 µM ABA) was not related to the contrasting levels of dormancy exhibited by sorghum lines RedlandB2 and IS9530. GA levels, in contrast, correlated with the expression of dormancy in sorghum grains. The embryo content of active GA_4_ in immature grains increased and reached a significantly higher value after a 4 d incubation period in the low dormancy line RedlandB2 as compared with the more dormant IS9530, and this increase occurred before embryo growth began (Perez-Flores *et al.*, 2003; Rodríguez *et al.*, 2012). It is precisely at this immature stage, before grain physiological maturity is achieved, that GA *de novo* synthesis contributes to germination of RedlandB2 seeds, as suggested by the results of Rodríguez *et al.* (2012).

Transcriptional analysis of several sorghum genes encoding putative GA synthesis enzymes (*SbEKO*, *SbEKAH*, *SbGA20ox2*, *SbGA20ox3*, and *SbGA3ox1*) showed a transient increase of these transcripts in dormant grains (IS9530) during the first 2–3 d of grain imbibition, but this did not occur in the less dormant genotype (RedlandB2) (Rodríguez *et al.*, 2012). This evidence appears to be in contradiction to changes in GA_4_ levels in both lines. However, this GA synthesis ‘intention’ in the more dormant IS9530 occurred together with an evident promotion of the GA inactivation genes *SbGA2ox1* and *SbGA2ox3*. This observation, together with a negative association between embryo content of active GA_4_ and its corresponding catabolite GA_34_, supported the idea that GA_4_ levels are kept low in dormant IS9530 grains as a result of a prominent catabolic activity by GA 2-oxidases (GA2oxs) which is not evident in RedlandB2 embryos. On the other hand, incubation of dormant grains in 100 μM GA_3_ promoted germination but did not reduce the expression of most key GA synthesis genes, ruling out the idea of a negative feedback regulatory mechanism driven by active GAs. In addition, and in contrast to other reports which show a feed-forward mechanism affecting expression of *GA2ox* genes by active GA levels in *Arabidopsis* (Ogawa *et al.*, 2003; Rieu *et al.*, 2008), expression of sorghum *GA2ox1* and *GA2ox3* was down-regulated by exogenously applied GA_3_ (Rodríguez *et al.*, 2012). The temporal coincidence of expression patterns for *SbABI4*, *SbABI5* (together with SbABI5 protein abundance), and *SbGA2ox* genes, coinciding also with impairment of GA_4_ accumulation and absence of germination, suggested ABA signalling as a candidate pathway for the regulation of the expression of *SbGA2ox1* and *SbGA2ox3* in dormant IS9530 grains (Supplementary Fig. S1 available at *JXB* online; Rodríguez *et al.*, 2009, 2012).

Evidence of interactions between ABA signalling and GA metabolism had not been provided until recently. Lee *et al.* (2012) reported that AtABI5 is capable of binding *AtGA3ox1* and *AtGA3ox2* promoters *in vivo* and repressing their expression during the blocking of phyB-dependent germination. However, a cross-talk mechanism between ABA signalling and GA metabolism during dormancy expression has not been addressed for a species with agronomical importance such as grain sorghum. The *in silico* analysis of the *SbGA2ox3* 5′-regulatory region carried out by Rodríguez *et al.* (2012) revealed the presence of several *cis*-regulatory elements related to ABA and GA signalling. In particular, elements such as the RY repeat, CE, and ABRE were found to be located close to the TATA-box and with a spatial configuration similar to that which had been previously reported to be required for ABA induction of other promoters (Himmelbach *et al.*, 2003; Shen *et al.*, 2004). These findings suggested the possibility that the GA metabolism gene *SbGA2ox3* could be regulated by some ABA signalling factors in immature dormant sorghum grains. In this regard, considering the synchrony of *SbABI4*, *SbABI5*, and *SbGA2ox3* expression profiles and the SbABI5 abundance pattern in incubated dormant sorghum grains, together with the identification of a possible ABRC located on the *SbGA2ox3* promoter, the possibility of a protein–DNA interaction between ABI4 and ABI5 proteins and the *SbGA2ox3* 5′-regulatory region was tested *in vitro* by performing electrophoretic mobility shift assays (EMSAs). The results show that both ABI5 and ABI4 can interact *in vitro* with a DNA fragment identical to a region of the *SbGA2ox3* promoter containing an ABRE and CE, although they were not able to bind this DNA fragment simultaneously.

Although further evidence is needed to confirm the occurrence of this interaction *in vivo*, a model is proposed where *SbGA2ox3* transcription could be enhanced by SbABI5 and/or SbABI4, increasing GA degradation, which would finally lead to the blocking of germination in dormant immature sorghum seeds. Furthermore, in order to explore whether this cross-talk scheme between ABA signalling and GA metabolism is conserved among different species, a phylogenetic analysis of *GA2ox* genes in monocot and dicot species was carried out, and the existence of conserved regulatory ‘complexes’ within their promoters was explored as evidence of probable functional importance. The ABRC first identified in the sorghum *GA2ox3* promoter region emerged as a highly conserved module among several monocot species, which was absent in the analysed dicots.

## Materials and methods

### *In silico* analysis of the *SbGA2ox3* promoter

The *SbGA2ox3* gene KEGG ID code (Sb03g035000) was used at Phytozome to identify a genomic sequence of 2kb extending 5′ from the translation start site of the *SbGA2ox3* gene. The 2kb sequence was considered a gene regulatory region or promoter as no introns were detected upstream of the translation start site of *SbGA2ox3*. The sequence was scanned for the presence of putative *cis*-regulatory elements at the PLACE database (http://www.dna.affrc.go.jp/htdocs/PLACE/).

### Plant material, RNA extraction, and cloning of *SbABI4* and *SbABI5*

IS9530 sorghum [*S. bicolor* (L.) Moench] inbred line was sown in the experimental field of the Institute for Agricultural Plant Physiology and Ecology (IFEVA) of the Faculty of Agronomy (Buenos Aires University). Anthesis date was recorded for each plant, and, at 30 d post-anthesis, seeds from eight plants were collected, pooled, and incubated in Petri dishes with distilled water at 25 °C. Embryos were dissected after 1, 2, and 3 d of incubation and stored in liquid N_2_. Samples of 30–45 embryos (70–100mg) were ground to powder using a mortar and pestle in liquid N_2_ and added to 600 µl of the RA1 extraction buffer included in the Nucleospin RNAII kit (Macherey-Nagel, Germany). PVP-40 (Sigma) was added to the RA1 buffer to a final concentration of 1% (wv). After 5min centrifugation, clear supernatant was used for the extraction protocol described in the kit’s manual. Reverse transcription was performed with M-MLV Reverse Transcriptase (Promega) and the resulting cDNA aliquots were pooled to use as PCR templates. *SbABI4* and *SbABI5* cDNA were cloned by PCR from these cDNAs with the addition of *Bam*HI and *Xho*I restriction sites in the following primers: *SbABI4*, ACGGGATCCGAACCCAACAACAATCAG (forward) and GCGCTCGAGCTTGAGGAAGACATCAAACC (reverse); *SbABI5*, GAGAGGATCCAATTTCCCGGGAGGAAGCG (forward) and GAGACTCGAGCCACGGACCTGTCAATGTC (reverse). The resulting PCR products were purified with a NucleoSpin Gel and PCR Clean-up kit.

### Expression and purification of recombinant SbABI4 and SbABI5 proteins

*SbABI4* and *SbABI5* purified cDNAs were ligated into the pGEM-T Easy vector (Promega) and used to transform DH5α *Escherichia coli* cells. Plasmids were purified by Wizard Plus Sv Minipreps DNA Purification Systems (Promega) and digested with *Bam*HI and *Xho*I. The digestion product was subsequently ligated into pET24a containing a C-terminal histidine tag, and Rossetta pLys *E. coli* cells were transformed with the recombinant plasmids. Bacteria were grown in LB medium with kanamycin and chloramphenicol overnight, and 10ml were used to inoculate 1 litre of fresh LB medium. Cultures were grown until OD_600_=0.6 and induced for 16h with 0.05mM isopropyl-β-d-thiogalactopyranoside (IPTG), at 20 ºC. The resulting pellet was sonicated in lysis buffer (25mM Tris-HCl pH 7.4, 300mM NaCl, and 30mM imidazole) and loaded onto a His GraviTrap column (GE Healthcare). Elution of recombinant protein was achieved in buffer containing 25mM Tris-HCl, 300mM NaCl, and 350mM imidazole, according to the kit instructions. Purified protein was stored at –80 ºC with addition of 5% sucrose and 3mM 2-mercaptoethanol.

### Electrophoretic mobility shift assays

*SbGa2ox3* probe was obtained by PCR, with a 5′-biotinylated primer (5′BIOT GGGCGCCGTGGGAAAACTG3′) and a 3′-non-biotinylated primer (5′GGGCGGCACCTGGCTGGATG3′), using genomic DNA from the IS9530 grain sorghum line as template. The resulting fragment (242bp long) was purified with NucleoSpin Gel and PCR Clean Up (Macherey Nagel). The specific competitor fragment was obtained by PCR with the same primers as the probe, but without the biotinylated 5′ end. Alternative shorter biotinylated probes for the *SbGA2ox3* promoter were generated by combining the same 5′-biotinylated primer and the following 3′-non-biotinylated primers: 5′GGGCGCGACGTGTCCGGACGCG3′ (131bp product, intact ABRE and CE); 5′GGGCGCGAATTGTC CGGACGCG3′ (131bp product, mutated ABRE), and 5′ACGCG ATCCACCGGAAGCAGG3′ (114bp product, mutated CE and no ABRE included). The non-specific competitor (183bp fragment) was amplified by PCR from the *SbGAMyb* gene. The *AtEm6* promoter probe (186bp) was also generated by PCR on *Arabidopsis* genomic DNA as template and with the following primers: 5′BIOT AGTTAAAGAACACGCGGCGA3′ and 5′TCAATCCGGAGGGCGTTTTGG3′. Complete sequences for all probes are included in Supplementary Fig. S2 at *JXB* online.

Binding reactions were performed with binding buffer [0.5mM EDTA, 3mM MgCl_2_, 14mM 2-mercaptoethanol, 10% (v/v) glycerol, 0.05% (v/v) NP-40, 10 µg ml^–1^ bovine serum albumin (BSA), 25mM TRIS-HCl pH 7.4, 1mM dithiothreitol (DTT), and 30mM KCl], 50 µg ml^–1^ salmon sperm DNA, 40ng of biotinylated probe, a variable amount of purified recombinant protein SbABI5 or SbABI4, and specific or non-specific competitor fragments (5×, 10×, or 20×). Incubations were carried out for 30min at room temperature and loaded on an 8% (for SbABI4 assays) or 6% (for SbABI5 assays) polyacrylamide gel (29:1 acrylamide:bisacrylamide). For specific and non-specific (*SbGAMyb*) probe competition assays, proteins were incubated with competitor probes for 10min prior to the addition of biotinylated probe and incubation was continued for an extra 20min. For the non-specific protein assays, total protein extract was obtained from induced cultures of pET24a vector without insert and used for shift assays, incubating the reactions for 30min. In all cases, gels were electrophoresed at 120V in 0.5× TBE and transferred to a nylon membrane (Amersham Hybond-N^+^). The resulting blots were blocked with TTBS–5% low fat milk for 2h, and incubated overnight with High Sensitivity Streptavidin HRP Conjugate (Thermo). Detection of bound streptavidin protein was performed with ECL substrate and CL-X Posure Film (Thermo).

### Identification of GA2ox sequences and phylogenetic analysis

In this work, the GA2ox full-length amino acid sequences of *Arabidopsis thaliana* (AtGA2ox), *Oryza sativa* (OsGA2ox), *Sorghum bicolor* (SbGA2ox), and *Zea mays* (ZmGA2ox) previously described by Rodríguez *et al.* (2012) were used. In order to expand the GA2ox family, the KEGG (http://www.genome.ad.jp/kegg/) nucleotide and protein sequence databases for fully sequenced genomes were scanned for GA2ox in the species *Medicago truncatula* (MtGA2ox), *Vitis vinifera* (VvGA2ox), *Populus trichocarpa* (PtGA2ox), and *Brachypodium distachyon* (BdGA2ox). Identification codes for sequences are listed in Supplementary Table S1 at *JXB* online. With these amino acid sequences, a set of GA2ox proteins represented in four monocot and four dicot species was completed. The alignment of the full-length amino acid sequences was performed in ClustalW (Thompson *et al.*, 1997) using standard settings (Gonnet weight matrix, gap opening=10 and gap extension=0.2) and was adjusted by visual inspection. Bayesian phylogenetic analyses on aligned full-length sequences were performed with MrBayes v. 3.1.2 setting an MCMC algorithm (Ronquist and Huelsenbeck, 2003). Two independent runs were computed for 1 500 000 generations (Supplementary Fig. S3). In addition, using the Neighbor–Joining (NJ) and maximum parsimony (MP) methods, trees with similar topology were obtained (data not shown). All trees were visualized using the program MEGA 5.01 (Tamura *et al.*, 2011). From the obtained tree (Supplementary Fig. S3), only the group including SbGA2ox3 (I) was selected and divided into subgroups D, M1, M2, and M3 for the following analysis. A 2kb fragment upstream of the ATG was identified for each gene as the putative regulatory region (promoter). The *SbGA2ox4* promoter was not included in the search, as the SbGA2ox4 hypothetical protein is truncated and probably non-functional. A comparative analysis using the 2kb promoter sequences was performed within each of four subgroups (D, M1, M2, and M3) using the EARS tool (http://wsbc.warwick.ac.uk/ears/help.php). EARS software breaks sequences into small subsequences (windows) and carries out global alignments between each possible pair of windows, allowing the detection of conserved sequences. Four independent EARS runs were carried out, comparing the *SbGA2ox3* promoter with D, M1, M2, and M3 GA2ox promoters, respectively. For all the runs, a windows size of 60bp and a cut off *P*-value of 0.0001 were used. The EARS result file for each run was analysed and, in the case of the M3 subgroup, the location of the significant peaks detected (1 and 2) within *SbGA2ox3* and M3 GA2ox promoters was established. Finally, sequences corresponding to peaks 1 and 2 from *BdGA2ox5*, *BdGA2ox8*, *OsGA2ox3*, *OsGA2ox4*, and *SbGA2ox3* promoters were run on MEME (Bailey *et al.*, 2009) to identify the conserved motifs represented on those fragments.

## Results

### *In silico* analysis of the *SbGA2ox3* 5′-regulatory region

**Fig. 1.**
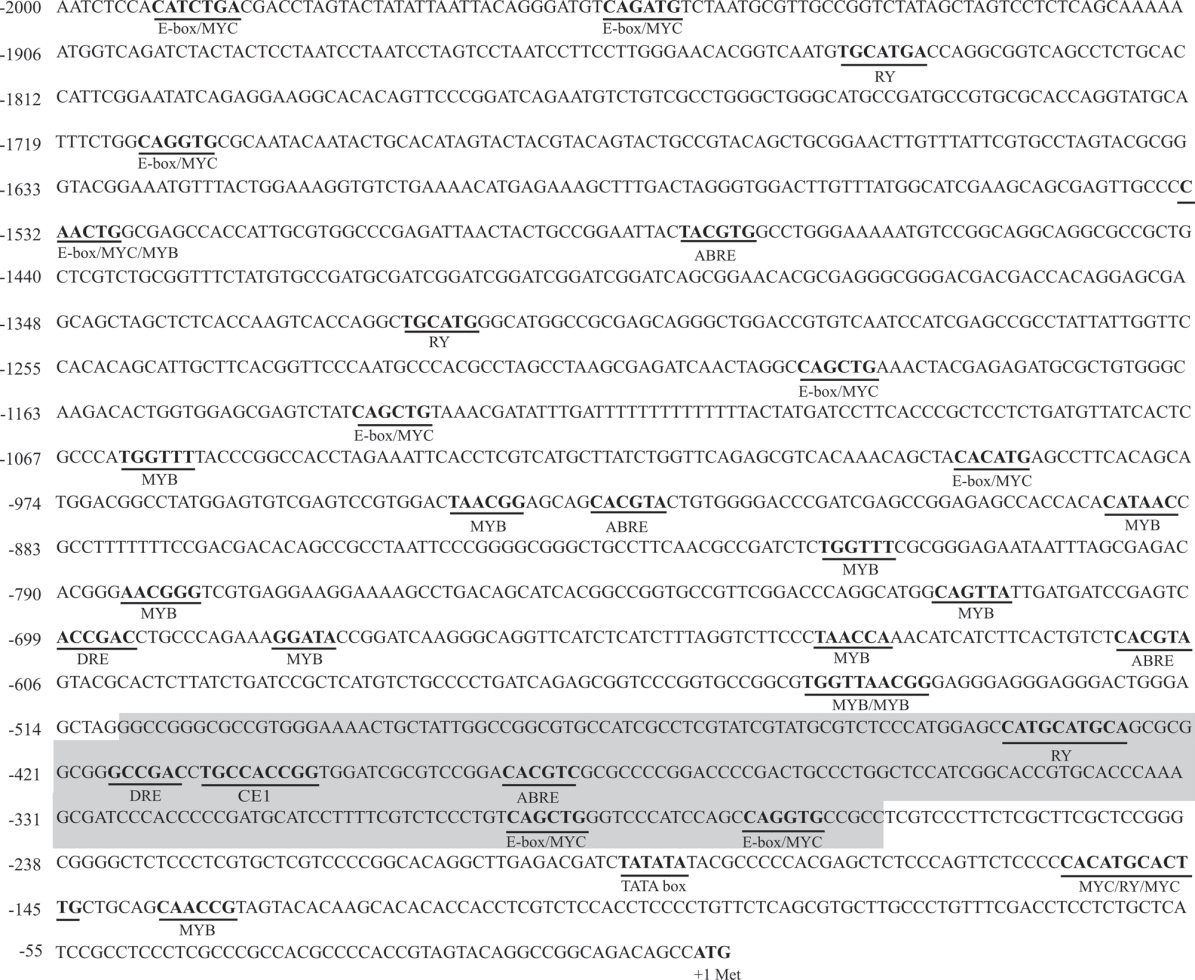
*SbGA2ox3* putative 5′-regulatory region sequence, comprising 2000bp upstream from the ATG (in bold). Positions are given relative to the first base of the initiating methionine. Potential transcription binding motifs ABRE, CE, DRE, RY, MYB, and E-box/MYC are underlined and named below. The 242bp biotinylated fragment used as probe in electrophoretic mobility shift assay (EMSA) experiments is highlighted in grey. (This figure is available in colour at *JXB* online.)

In previous work (Rodríguez *et al.*, 2012), several regulatory motifs related to ABA signalling were found within the first 557bp upstream from the transcription initiation site of the *SbGA2ox3* gene. In order to broaden this analysis, and taking into account that many authors have reported the occurrence of plant transcription factors binding *cis*-elements located at 2kb or 3kb upstream from the ATG (Kim, 2007; Oh *et al.*, 2007; Lee *et al.*, 2012), a genomic sequence of 2kb, considered to be the putative 5′-regulatory region (promoter), was cloned and sequenced for both sprouting-resistant (IS9530) and sprouting-susceptible (RedlandB2) genotypes. To assess the possibility of a differential sequence composition in the regulatory region between the sprouting-resistant and sprouting-susceptible lines, both sequences were analysed in the PLACE database to identify *cis*-acting regulatory elements. Several *cis*-acting regulatory sequences were identified, including previously reported ABA and GA signalling elements, with ABRE, CE, DRE (drought response element), RY repeat, MYB, E-box/MYC, and GA-down (similar to ABRE), being present at the highest density of these regulatory sequences found within the first 1000bp upstream from the ATG (Fig. 1). Among the ABA response group, an ABRE, a CE1, and a DRE motif were found to be located at positions –194, –218, and –225bp upstream of the TATA-box, respectively. This particular succession of regulatory elements has been previously reported by other authors to be present in the promoters of ABA-regulated genes (Himmelbach *et al.*, 2003; Shen *et al.*, 2004) and could be considered as an ‘ABA response complex’ (ABRC) on the *SbGA2ox3* promoter. On the other hand, many RY and E-box/MYC elements were also found in the analysed region, and it has been reported that these motifs frequently occur in the 5′-regulatory sequence of genes that exhibit seed-specific expression (Stalberg *et al.*, 1996; Ezcurra *et al.*, 1999; Kim *et al.*, 2007). In particular, the location of one of the RY sequences, at position –244bp upstream of the TATA box, and near the possible ABRC, suggested possible promoter seed-specific regulation by ABA for *SbGA2ox3*. These findings are in agreement with several studies that have shown that regulatory elements conferring seed-specific expression appear in the proximal region of the promoter, often within 500bp upstream of the transcriptional start (Wu *et al.*, 2000; Chandrasekharan *et al.*, 2003).

The complete analysis of the 5′-regulatory region allowed the definition of a region within the promoter as a candidate sequence to be bound by SbABI4 and SbABI5. According to this analysis, the region located from –505bp to –263bp upstream from the ATG contained a putative ABRC (ABRE and CE1), a possible target for both transcription factors (ABI4 and ABI5). Therefore, this was precisely the fragment that it was decided to use as a probe in the first place for the subsequent EMSAs (Fig. 1).

When comparing the 2kb promoter region for *SbGA2ox3* from both RedlandB2 and IS9530, some point mutations were detected, and only one base was different within the –505 to –236 sequence (probe region) which did not affect any of the ABRC elements (Supplementary Fig. S4 at *JXB* online). So far, considering *cis*-regulatory elements involved in ABA signalling, no differences were found in the *SbGA2ox3* promoter region from both lines that could be related to the observed contrasting expression of this gene.

### SbABI4 and SbABI5 bind to the *SbGA2ox3 in vitro*

**Fig. 2.**
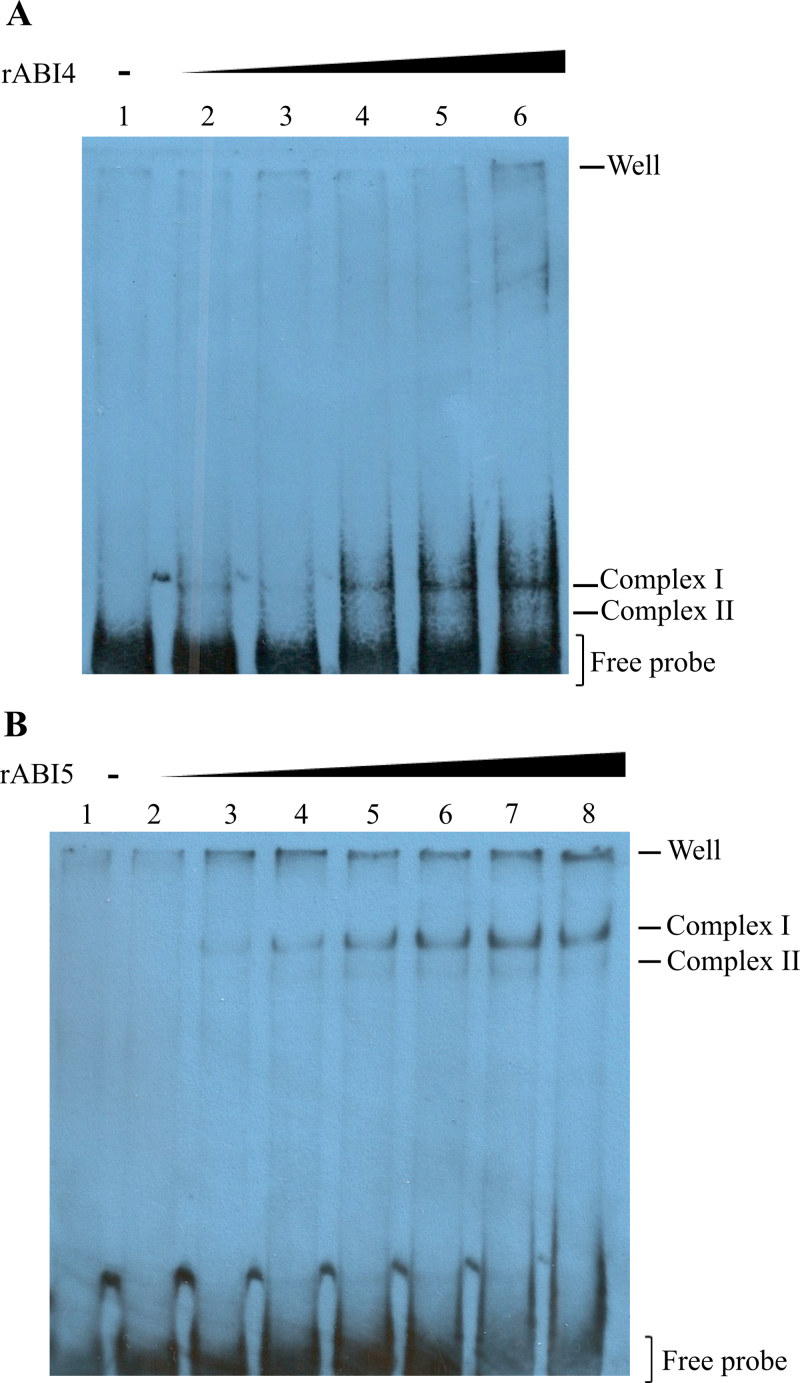
DNA binding activity of recombinant *S. bicolor* ABI4 and ABI5 (rABI4 and rABI5). EMSA experiments were performed with 40ng of a 242bp (–505bp to –263bp) *SbGA2ox3* promoter biotinylated probe and increasing amounts of recombinant proteins rABI4 and rABI5. Complexes detected, well position, and free probe are indicated. (A) Band shift pattern for rABI4. The protein amount in each reaction was as follows: lane 1, no protein; lane 2, 0.045 μg; lane 3, 0.075 μg; lane 4, 0.15 μg; lane 5, 0.225 μg; lane 6, 0.3 μg. (B) Band shift pattern for rABI5. The protein amount in each reaction was as follows: lane 1, no protein; lane 2, 0.03 μg; lane 3, 0.06 μg; lane 4, 0.15 μg; lane 5, 0.3 μg; lane 6, 0.375 μg; lane 7, 0.47 μg; lane 8, 0.6 μg. (This figure is available in colour at *JXB* online.)

To test whether SbABI4 and SbABI5 can bind the *SbGA2ox3* promoter region containing the putative ABRC, both *S. bicolor* cDNA fragments were cloned from IS9530 and both proteins were expressed with a C-terminal His-tag. The ability of recombinant ABI4 (rABI4) and rABI5 to bind the *SbGA2ox3* promoter was analysed by EMSAs using the biotinylated –505bp to –263bp probe. Incubation of this probe with 0.135 μg of rABI4 led to the formation of two complexes (Fig. 2A), suggesting the presence of two binding sites for ABI4 in the *SbGA2ox3* probe. Similar results were obtained when incubating the probe with purified rABI5: two major bands were observed using 0.75 μg of protein, indicating that the transcription factor can bind the probe at two sites (Fig. 2B). The slowest migrating complex I can be related to the transcription factor binding two *cis*-elements simultaneously, and the faster migrating complex II results from the probe being bound at only one of the *cis*-regulatory elements. For both transcription factors ABI4 and ABI5, complex I (double binding of the probe) showed a stronger signal than did complex II with all protein concentrations, indicating a lower probability of the single bound form in the *in vitro* conditions used here. When comparing EMSA results for ABI4 and ABI5 assays, the amount of protein that led to the formation of both complexes in each case was quite different. In this sense, a 5-fold higher amount of rABI5 was needed to detect the retarded complexes, as compared with the amount of rABI4. This can be ascribed in part to the fact that the SbABI5 functional form is a dimer of 82kDa while SbABI4 is 30kDa and binds as a monomer (Nakamura *et al.*, 2001); hence, to obtain an equal number of ‘binding units’, a minimal 2.7-fold SbABI5 protein is required as compared with SbABI4, assuming a 100% efficiency of SbABI5 dimerization under the *in vitro* conditions used here.

**Fig. 3.**
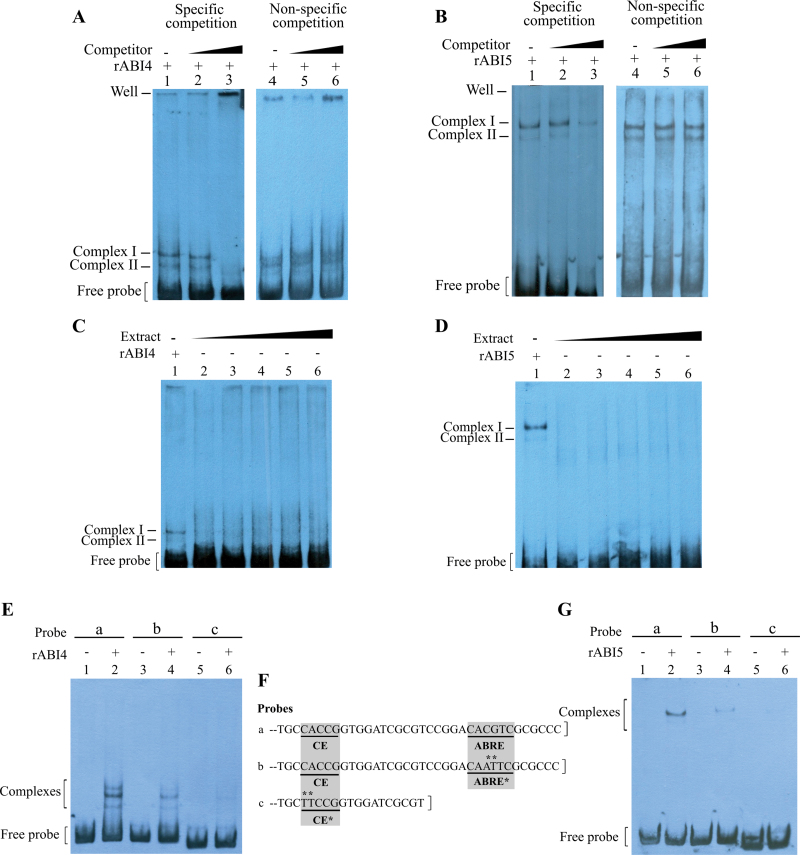
(A and B) Competition EMSAs for rABI4 and rABI5 performed with a 242bp *SbGA2ox3* fragment (–505bp to –263bp) as biotinylated probe. For all lanes, 40ng of biotinylated probe and 0.225 μg of rABI4 (A) or 0.375 μg of rABI5 (B) were used. For specific competitions, the mass of unlabelled competitor DNA (*SbGA2ox3* unlabelled fragment) in each reaction was as follows: lane 1, no competitor; lane 2, 200ng; lane 3, 600ng. For non-specific competitions, the mass of unlabelled competitor (*SbGAMyb* 183bp fragment) was as follows: lane 4, no competitor; lane 5, 200ng; lane 6, 600ng. (C and D) Control EMSAs for rABI4 (C) and rABI5 (D) performed with increasing concentrations of protein extract from *E. coli* cultures (transformed with empty pET24a) and 40ng of *SbGA2ox3* biotinylated probe. For both C and D, the protein extract amount in each lane was as follows: lane 2, 10.74 μg; lane 3, 14.32 μg; lane 4, 17.9 μg; lane 5, 21.48 μg; lane 6, 25.06 μg. Lane 1 shows the positive control incubation reaction with 40ng of *SbGA2ox3* biotinylated probe and 0.225 μg of rABI4 (C) and 0.375 μg of rABI5 (D). (E and G) EMSAs carried out with rABI4 (E) or rABI5 (G) and shorter *SbGA2ox3* probes a, b, and c. For all lanes, 40ng of biotinylated probe was used. The protein amount was as follows: lanes 1, 3, and 5, no protein; lanes 2, 4, and 6, 0.225 μg of rABI4 (E) and 0.375 μg of rABI5 (G). (F) Sequences of probes a, b, and c with highlighted CEs and ABREs. Probe a, wild-type probe (intact ABRE and CE); probe b, mutated ABRE; probe c, mutated CE and no ABRE included. Asterisks in probes b and c indicate mutated bases and elements. Probe size was 131bp for a and b, and 114bp for c. (This figure is available in colour at *JXB* online.)

To test the specificity of the binding of rABI4 and rABI5 to the *SbGA2ox3* probe, several gel shift experiments were carried out using non-biotinylated specific and non-specific probes or non-specific protein extracts. Competition with increasing concentrations of the specific unlabelled probe of *SbGA2ox3* completely displaced complexes I and II for both rABI4 and rABI5 incubations, whereas competition with the unlabelled fragment of non-specific probe (*SbGAMyb*) had no effect (Fig. 3A, B). To rule out a possible interaction between residual *E. coli* proteins and the probe used, incubation reactions were performed with variable amounts of crude protein extracts from *E. coli* transformed with empty pET24a; these incubations did not lead to the formation of any complex (Fig. 3C, D).

**Fig. 4.**
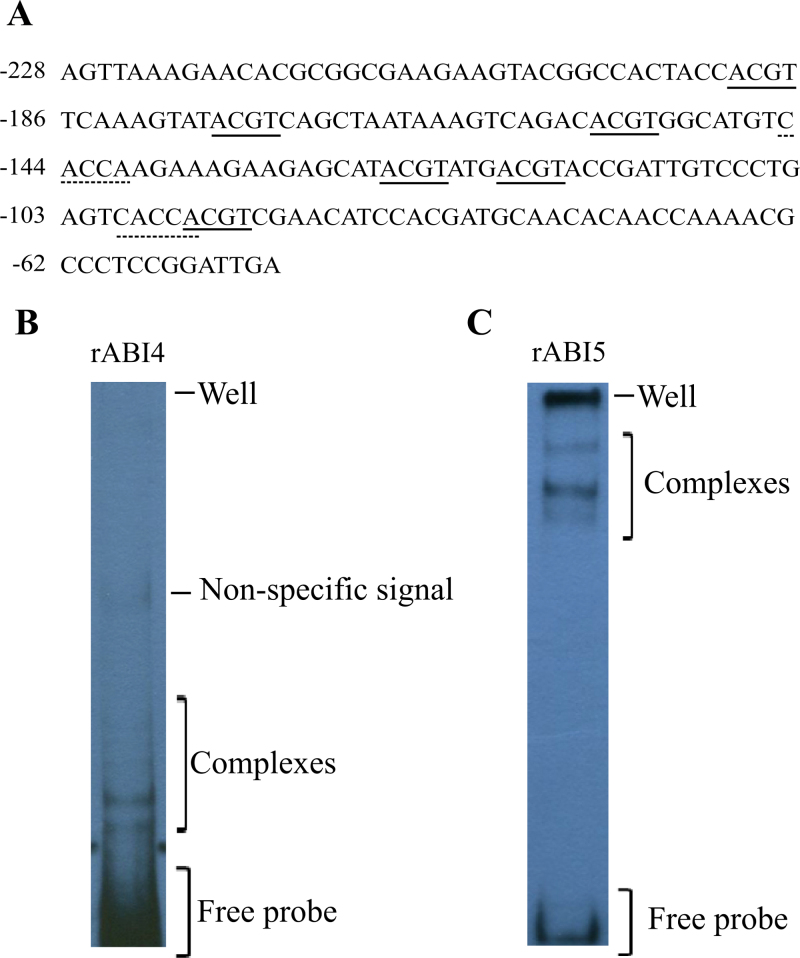
rABI4 and rABI5 binding affinity for an *AtEm6* promoter biotinylated probe. (A) *AtEm6* biotinylated probe sequence (186bp). Positions are given relative to the first base of the initiating methionine (ATG). ACGT cores for putative ABREs (solid lines) and CE-like (dashed lines) motifs are indicated. (B) Band shift pattern for rABI4. A 40ng aliquot of *AtEm6* biotinylated probe was incubated with 0.225 μg of rABI4. (C) Band shift pattern for rABI5. A 40ng aliquot of *AtEm6* biotinylated probe was incubated with 0.375 μg of rABI5. In all cases, complexes detected, well position, and free probe are indicated. (This figure is available in colour at *JXB* online.)

In order to examine the possibility that the ABRC included in the *SbGA2ox3* 5′-regulatory region was involved in the detected binding to both rABI4 and rABI5 and to discard possible artefacts due to the probe length used, shorter biotinylated probes were designed, including a wild-type sequence (with an intact ABRC), a mutated ABRE probe (with a mutated ABRE and intact CE), and a mutated CE probe (with a mutated CE and without the ABRE) (Fig. 3F). When EMSA experiments were performed with the wild-type sequence probe, both rABI4 and rABI5 produced similar complexes to those with the larger probe, with the appearance of an additional retarded complex in the case of rABI4. However, when rABI4 or rABI5 was incubated with the mutated ABRE probe, the intensity of all complexes decreased, indicating a lower affinity for that DNA fragment. Moreover, when the mutated CE probe was used (and no ABRE was included in this case), only traces of a retarded complex were detected, suggesting that the affinity for this probe was even weaker (Fig. 3E, G). On the other hand, positive control shift assays were performed incubating rABI4 or rABI5 with a biotinylated *AtEm6* promoter probe, that contains six putative ACGT cores that could act like ABRE and two CE-like elements (Fig. 4A), and that had been previously demonstrated to be bound by AtABI5 (Carles *et al.*, 2002). Both rABI4 and rABI5 were capable of binding the *AtEm6* probe, giving rise to five and four retarded complexes, respectively (Fig. 4B, C). These results suggest that ABRE and CE have a decisive role in the binding of rABI4 and rABI5 to the *SbGA2ox3* promoter. Taken together, the results of competition assays and positive and negative control experiments demonstrate that *in vitro* binding of rABI4 and rABI5 to the *SbGA2ox3* probe occurs in a specific manner.

### SbABI4 and SbABI5 do not bind simultaneously to the *SbGA2ox3 in vitro*

**Fig. 5.**
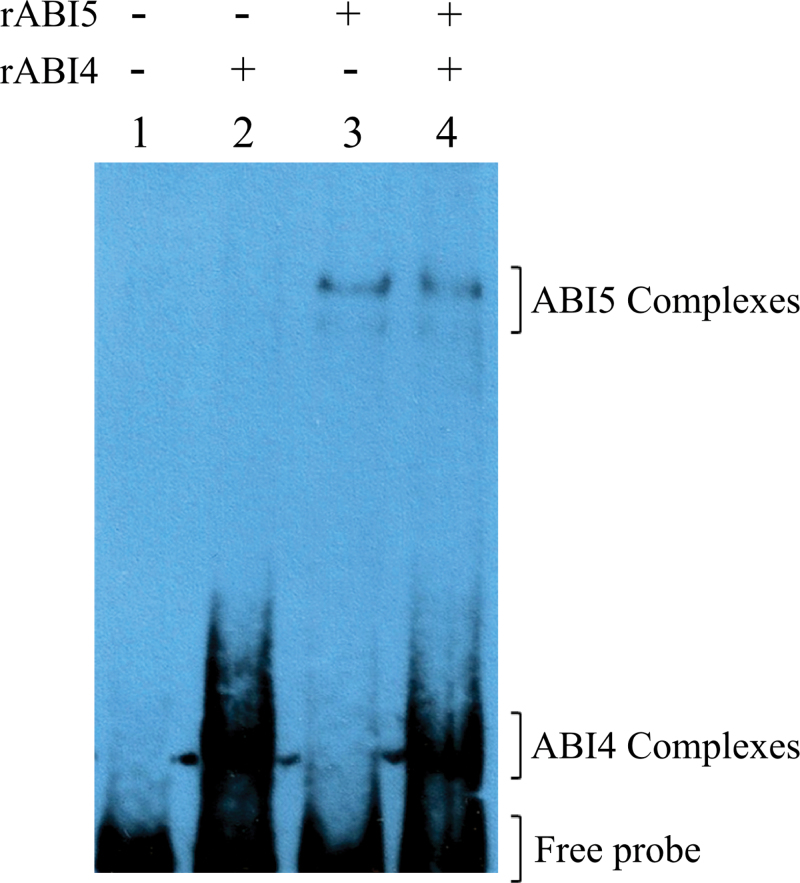
EMSA performed with rABI4 and rABI5 co-incubations with 40ng of *SbGA2ox3* biotinylated probe. Protein amounts in the reactions were as follows: lane 1, no protein; lane 2, 0.225 μg of rABI4; lane 3, 0.375 μg of rABI5; lane 4, 0.225 μg of rABI4 and 0.375 μg of rABI5. Complexes and free probe are indicated. (This figure is available in colour at *JXB* online.)

Considering that both SbABI4 and SbABI5 bound to the same *SbGA2ox3* 5′-regulatory region and that it has been recently reported that ABI4 and ABI5 could synergistically activate some plant promoters (Reeves *et al.*, 2011), the possibility that ABI4 and ABI5 recombinant proteins could bind simultaneously to the same *SbGA2ox3* promoter probe was examined. Contrary to this hypothesis, shift assays performed by co-incubating the *SbGA2ox3* probe with both proteins did not generate any ternary complex, suggesting that SbABI4 and SbABI5 do not bind to the *SbGA2ox3* promoter simultaneously (Fig. 5). Hence, it is possible that they have affinity for the same regulatory elements.

### ABRC is found in the promoters of other monocot *GA2ox* genes

**Fig. 6.**
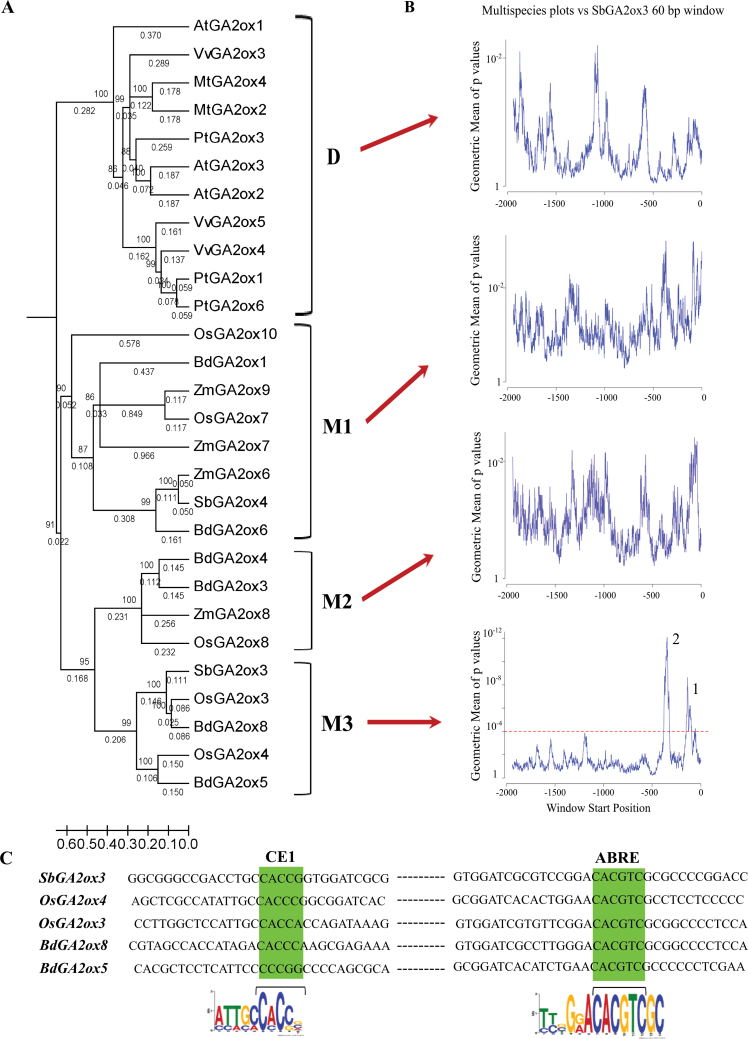
Comparative analysis of regulatory regions of GA2ox promoters. (A) Phylogenetic relationships for group I GA2oxs. The 28 GA2ox proteins were grouped into four subgroups: D, dicots; M1, monocots 1; M2, monocots 2; and M3, monocots 3. At, *Arabidopsis thaliana*; Bd, *Brachypodium distachyon*; Mt, *Medicago truncatula*; Os, *Oryza sativa*; Pt, *Populus trichocarpa*; Sb, *Sorghum bicolor*; Vv, *Vitis vinifera*; Zm, *Zea mays*. Bootstrap support values are indicated and scale bars specify the number of changes per position for a unit branch length. Identification codes for sequences are listed in Supplementary Table S1 at *JXB* online. (B) Multispecies plots showing comparative analysis of the *SbGA2ox3* promoter versus promoters of each subgroup (D, M1, M2, and M3), performed with the EARS tool. The red dashed line indicates the selected cut-off *P*-value (0.0001), suggesting that only in the case of M3 were two significant peaks detected. (C) Sequence alignments of significant peak sequences of M3 members (*SbGA2ox3*, *OsGA2ox3*, *OsGA2ox4*, *BdGA2ox5*, and *BdGA2ox8*). Conserved regions are highlighted in green: ABRE (CACGTC) and CE (CACCG). The MEME software (Bailey and Elkan, 1994) was used to find common motif logos in the promoters of subgroup M3 members. CE and ABRE motif logos are shown below each conserved sequence.

In view of the *in vitro* results obtained by EMSAs, the next objective was to investigate whether the interactions detected are part of a conserved regulatory mechanism of *GA2ox* genes in other species. Thus, the possibility that those GA2oxs with high sequence similarity to SbGA2ox3 would share the same regulatory elements in the 5′-regulatory region was tested. To address this question, a global phylogenetic analysis was first conducted using the whole set of described GA2oxs involved in GA metabolism from four dicot and four monocot species. The full-length GA2ox protein sequences from these eight species were completely aligned and a phylogenetic tree was obtained (Supplementary Fig. S3 at *JXB* online) using MrBayes (Huelsenbeck and Ronquist, 2001). Taking into account the tree topology structure, branch lengths, and clade support values, GA2ox proteins were classified into three groups: I, II, and III, corroborating the classification proposed by Lee and Zeevart (2005) and the specific results reported by Rodríguez *et al.* (2012). SbGA2ox1 and SbGA2ox2 were included in group II, while SbGA2ox3 and SbGA2ox4 were members of group I, and, as the main interest of this study was focused on SbGA2ox3, group I was selected for further analyses. A clear separation between monocot (M) and dicot (D) GA2oxs was observed within group I, and the M group was further divided into M1, M2, and M3 subgroups (Fig. 6A). When the motif composition of all monocot GA2oxs was examined (Supplementary Fig. S5), SbGA2ox4 appeared as a truncated and probably non-functional protein, almost certainly originating from a recent duplication of the *SbGA2ox3* gene.

To test the hypothesis of a conserved regulatory mechanism involving the *GA2ox* gene promoter, a comparative analysis of 5′-regulatory sequences (2kb upstream of the ATG) of *SbGA2ox3* against each subgroup promoter (i.e. D, M1, M2, and M3) was performed, with the aid of the EARS tool (Picot *et al.*, 2010). Results obtained from these analyses showed that only promoters of *GA2ox* genes within the M3 subgroup share common regions with the 5′-regulatory sequence of *SbGA2ox3*. Two peaks with high significance levels were obtained, indicating the existence of two conserved regions within these promoters (Fig. 6B). Additionally, pairwise comparisons (i.e. *SbGA2ox3* and each of the other *GA2ox* promoters within group M3) were performed, and it was found that the five M3 members shared the same two significant peaks (Supplementary Fig. S6 at *JXB* online). Then, with the aim of identifying the common motifs within these five *GA2ox* genes, the sequence composition of the two conserved regions was explored in detail. Peak 1 co-localized in all cases with the TATA-box, while peak 2 could be delimited between –460bp and –375bp upstream from the ATG of the *SbGA2ox3* promoter. The sequence corresponding to that second peak comprised a portion of the probes that had been previously used in the EMSAs described above, and the ABRE and CE1 were represented in all cases. The CE1 sequence comprised point mutations for both *Brachypodium GA2ox* promoters, compared with the CE1 described in other species such as maize, which would lead to CE1-like elements (Niu *et al.*, 2002). In all cases, the location of ABRE and CE1 with respect to the TATA-box was as had been previously described for ABRCs (Shen and Ho, 1995; Shen *et al.*, 1996), supporting a possible transcriptional regulation by the ABA pathway for these genes (Fig. 6C). Moreover, although not included in the conserved regions detected in the present analysis (due to greater variation in the positioning of these elements), RY repeats were represented in all M3 subgroup promoters, indicating that the possible ABA induction of these genes is seed specific. Taken together, the results from the phylogenetic and comparative analyses indicate that M3 subgroup GA2oxs not only show a structural and probable functional similitude, but also might share a common transcriptional regulatory mechanism. Transcriptional factors ABI4 and ABI5 involved in ABA signalling emerge as strong candidates to be part of that mechanism.

## Discussion

ABA signalling and GA metabolism are known to play a main role during dormancy expression in immature sorghum grains (Perez-Flores *et al.*, 2003; Rodríguez *et al.*, 2009, 2012). Previous results with the sorghum system used here (RedlandB2, low dormancy; and IS9530, high dormancy) indicated that embryo sensitivity to ABA was related to dormancy expression in these two genotypes (Steinbach *et al.*, 1995; Gualano *et al.*, 2007; Rodríguez *et al.*, 2009). Similarly, Rodríguez *et al.* (2009) found that sorghum genes encoding putative ABI3/VP1, ABI4, ABI5, and PKABA1 (positive regulators of ABA signalling) were highly expressed during the incubation of IS9530 dormant immature grains, but not in the less dormant genotype RedlandB2. In addition, the content of active GA_4_ reached a significantly higher value in less dormant RedlandB2 embryos during day 4 of grain incubation as compared with the more dormant IS9530 (Perez-Flores *et al*., 2003; Rodríguez *et al.*, 2012). A possible explanation for this differential GA_4_ accumulation was proposed by Rodríguez *et al.* (2012) who reported an increase in transcript levels for genes encoding putative GA synthesis enzymes in both dormant and non-dormant grains, but this was also accompanied by an evident promotion of the GA inactivation genes *SbGA2ox1* and *SbGA2ox3* only in dormant IS9530. A negative correlation between GA_4_ and the GA_34_ catabolite also supported an active role for GA catabolism in determining GA_4_ values during the expression of dormancy in these sorghum lines. Considering the coordinated expression of *SbABI4*, *SbABI5*, and *SbGA2ox3* and the SbABI5 accumulation profile in incubated immature dormant sorghum grains (Supplementary Fig. S1 at *JXB* online; Rodríguez *et al.*, 2009, 2012), together with the finding of a possible ABRC in the *SbGA2ox3* promoter, an interaction between SbABI4, SbABI5, and the *SbGA2ox3* 5′-regulatory region was examined *in vitro*.

In this study, it is demonstrated that SbABI4 and SbABI5 recombinant proteins are both capable of interacting *in vitro* with a fragment of the *SbGA2ox3* 5′-regulatory region, suggesting a cross-talk between ABA signalling and GA metabolism during imbibition of immature, dormant sorghum seeds. Moreover, the results suggest that these interactions could be part of a transcriptional regulatory mechanism particular to a group of monocot GA2oxs, that has not been described previously.

*In silico* analysis of the *SbGA2ox3* promoter revealed the presence of many *cis*-acting elements including several known to be related to ABA or GA signalling and seed-specific expression (Fig. 1). Of particular interest are the ABRE and CE1 motifs located close to the TATA-box that, together, constitute an ABRC. ABA-responsive elements including ABRE and CE have been previously identified in the promoters of the *HVA1* Lea gene (Shen *et al.*, 1996), ABA-induced *HVA22* (Shen and Ho, 1995), wheat *Em* (Guiltinan *et al.*, 1990), and rice genes *Rab16b* (Ono *et al.*, 1996), *Rab17* (Busk *et al.*, 1997), *Rab28* (Busk and Pagès, 1998), and *OsEm* (Hattori *et al.*, 1995).

In this study it has been demonstrated that both transcription factors, SbABI4 and SbABI5, are capable of binding the *SbGA2ox3* probe in a specific manner, generating two complexes; this indicates the existence of two binding sites within this region of the *SbGA2ox3* promoter for each protein (Fig. 2). Although further experiments, such as DNA footprinting, are needed to elucidate the regulatory motifs that are bound by these proteins, shift assays performed with mutated ABRE or mutated CE probes demonstrated that both ABRE and CE elements are required for specific binding of SbABI4/SbABI5 to the *SbGA2ox3* promoter, as the binding to the *SbGA2ox3* probe was markedly reduced when ABRE and CE sequences were mutated. On this same theme, according to what had previously been reported in maize (Niu *et al.*, 2002), a candidate element in the *SbGA2ox3* promoter to be possibly bound by ABI4 would be the CE1, located –218bp upstream from the TATA-box. On the other hand, the ABRE (located –194bp upstream from the TATA-box) emerges as an additional candidate motif to be recognized by ABI4 as probe binding was reduced when the ABRE was mutated. This is also supported by the results of shift assays performed with the *AtEm6* promoter probe containing six putative ABRE and two CE-like motifs, which gave rise to five complexes when incubated with rABI4. Moreover, Wind *et al*. (2012) proposed a model that predicts the binding of ABI4 to the G-box element, which is coincident with the strong ABRE sequence (CACGTG).

According to the existing information (reviewed in Shinozaki *et al.*, 2007) and the present EMSA results obtained with the *AtEm6* probe, ABI5 might bind the ABRE on the *SbGA2ox3* regulatory region generating one of the complexes detected, and the CE appears as a possible candidate to be recognized by ABI5 and to give rise to the additional complex. In this sense, Casaretto *et al.* (2003) reported that the barley bZIP HvABI5 recognized both the ACGT-box and the CE3 of ABRC3, suggesting the possibility that SbABI5 binds both ABRE and CE. In the same vein, although both rABI4 and rABI5 were capable of binding the *SbGA2ox3* promoter, they were not able to interact simultaneously with the biotinylated probe, suggesting that both proteins might compete for the same binding motifs. This is in accordance with results reported by Cassaretto *et al*. (2003) as described above and also with results of Reeves *et al.* (2011), who analysed the transcriptome of ABI4- and ABI5-overexpressing *Arabidopsis* plants and reported that the promoters of many target genes of ABI4 and/or ABI5 are enriched in ABRE but not in CE motifs. In this last work, Reeves *et al*. also reported binding of *Arabidopsis* ABI4 to a DNA probe containing only ABRE but no CE motifs.

The present results indicate that SbABI4 is required in smaller quantities than SbABI5 to detect retarded complexes. Part of this difference could be related to the fact that ABI5 is known to act as homodimer or heterodimer (Finkelstein and Lynch, 2000) and, on the other hand, it has been shown that phosphorylation stabilizes ABI5 and enhances its activity *in vivo* (Lopez-Molina *et al.*, 2001; Piskurewicz *et al.*, 2008), which would also lead to larger amounts of SbABI5 needed to detect *in vitro* binding. Taking into account that both SbABI4 and SbABI5 have the capability of binding the *SbGA2ox3* promoter, both transcription factors could be part of a mechanism of fine-tuning the expression of *SbGA2ox3* in imbibed dormant grains. Although SbABI4 expression has already been measured by Rodríguez *et al.* (2009), further experiments are needed to quantify SbABI4 protein abundance during the incubation of dormant grains in order to have a more accurate picture of SbABI4 accumulation during imbibition. Moreover, SbVP1 appears to be another strong candidate to regulate *SbGA2ox3* expression, according to Himmelbach *et al.* (2003), whose work suggested that the transcriptional modulation of ABA-regulated genes might include the combined action of bZIP, AP2, and B3 domain transcription factors. In this sense, although ABI3 does not interact directly with ABREs or CEs, it is able to bind RY elements and, in combination with closely located ABREs or CEs, could act as a transcription enhancer of seed-specific ABA-regulated genes (reviewed by Holdswoth *et al.*, 2008). In this context, the RY elements identified in the *SbGA2ox3* promoter reinforce the possibility of SbAVP1/ABI3 acting as an accessory enhancer of *SbGA2ox3* transcription in the seed.

Taken together, these results allow the proposal that during dormancy expression in dormant immature seeds, SbABI4 and/or SbABI5 (with the possible accessory action of SbVP1) might interact with the *SbGA2ox3* promoter, enhance its transcription, and lead to SbGA2ox3 protein accumulation; this would result in subsequent active GA degradation, thus preventing germination of dormant grains. Conversely, ABA signalling components in non-dormant immature grains are scarcely accumulated during imbibition, and the *SbGA2ox3* promoter could not be activated by SbABI5 and/or SbABI4; this would lead to the accumulation of active GA (due to weak inactivation) and, as a result, enhanced germination. Similarly, Lee *et al.* (2012) reasoned that during phyB-dependent inhibition of germination in *Arabidopsis*, AtABI5 interacts with *AtGA3ox1* and *AtGA3ox2*, but suppresses their expression instead, which results in lower GA levels in the seed.

The phylogenetic and domain architecture analyses demonstrated that SbGA2ox3 is probably the only functional GA2ox within group I, as SbGA2ox4 appeared as a truncated duplication of SbGA2ox3. The probable lack of functionality of SbGA2ox4 highlights the central biological role of SbGA2ox3 in grain sorghum. In this same vein, the phylogenetic analysis and protein alignments performed showed that SbGA2ox3 shares not only a conserved structure/function with other M3 subgroup members, but also a common transcriptional regulation. No conserved regulatory complexes were observed for the remaining subgroups D, M1, and M2. The results demonstrate that the members of the M3 GA2ox subgroup have two conserved sequences, one of them including both the ABRE and CE (ABRC). The functionality of *BdGA2ox5* the CE1-like element has not been tested in other promoters, and so it cannot be considered as a functional element until new evidence is reported, but the CE1-like element detected in the *BdGA2ox8* promoter has been shown to be functional for ABI4 binding in maize *ABI4*, *RAB28*, and *RAB17* and in barley *HVA22* and *HVA* (Niu *et al.*, 2002). These findings suggest that M3 GA2oxs could be ABA-regulated genes and that the ABA signalling and GA metabolism cross-talk proposed for grain sorghum could also operate in rice and *Brachypodium*, through the transcriptional regulation of *OsGA2ox3*, *OsGA2ox4*, *BdGA2ox5*, and *BdGA2ox8*. Furthermore, the finding of RY elements in the promoter of these four genes indicates that the regulation of ABA transcription could be also mediated by VP1/ABI3 in a seed-specific manner.

Along the same lines, a comparative analysis of ABA content on *Brachypodium* grains showed that although low dormant and high dormant genotype seeds that went through after-ripening had less ABA than dormant grains after 4 d of imbibition, ABA content in dry seeds from the less dormant genotype was higher than in seeds of the high dormant genotype. These results suggest that ABA levels by themselves cannot predict dormancy in different *Brachypodium* genotypes, and ABA sensitivity and other hormones such as GA could be playing an important role (Barrero *et al.*, 2012). Studies of transcript levels of GA metabolism genes or embryo sensitivity to ABA have not been reported yet for *Brachypodium*, so a possible role for these components in dormancy expression cannot be ruled out. On the other hand, it has been proposed that ABA content during imbibition and seed development of weedy red rice (*Oryza sativa*) is not directly linked to the dormancy level. Instead, ABA sensitivity seems to be an important component for dormancy status (Gianinetti *et al.*, 2007). Taken together, these results suggest that the ABA–GA cross-talk proposed for sorghum could also take place in rice and *Brachypodium* dormant seeds.

Although additional assays such as ChIP-PCR are necessary to confirm the *in vivo* occurrence of these interactions in the seed embryo, *in vitro* specific binding is the first proof required to clarify whether an interaction is possible or not. Current efforts are oriented towards studying the relevance of these interactions *in vivo* in sorghum immature, dormant grains, and identifying other potential transcriptional factors interacting with the *SbGA2ox3* promoter region in one-hybrid yeast assays. As mentioned before, the first evidence of an interaction between ABA signalling and GA metabolism has recently been reported by Lee *et al.* (2012) in the model species *A. thaliana*, but similar interactions have not been described yet for any species of agronomic relevance.

To conclude, even though GA metabolism enzymes are highly conserved in their structure and probably in their function, the regulation of these genes across different species presents high variability. The identification of regulatory steps in species with agronomic relevance is crucial for the planning of breeding strategies. Sorghum lines IS9530 and RedlandB2 present an experimental system based on intraspecific variability for the level of dormancy and PHS response. The present work offers new insights into the regulation of a sorghum GA catabolism gene which in previous works emerged as a strong candidate to regulate active GA_4_ levels and the germination response. The contrasting expression pattern of the *GA2ox3* gene in both lines is likely to rely on the differential activity of ABA pathway elements such as ABI4 and ABI5 rather than variability in the *GA2ox3* promoter sequence. In this sense, activation of GA catabolism by ABA signalling factors could be interpreted as an additional factor that contributes to the inhibition of germination as the ABA/GA balance is pushed further towards the action of ABA. It would be interesting to find out if this mechanism is present in other monocots in which ABA sensitivity, rather than ABA metabolism, is in control of the level of dormancy. Further studies with TILLING mutants from sorghum, *Brachypodium*, or rice affected in the *GA2ox* genes that harbor the ABRC in their promoters might prove useful to understand the contribution of this gene to the expression of dormancy.

## Supplementary data

Supplementary data are available at *JXB* online.

Figure S1. (A) Embryonic transcript levels for *SbABI4*, *SbABI5*, and *SbGA2ox3* genes during incubation at 20 ºC of immature grains of RedlandB2 and IS9530. (B) Evolution of germination percentage and embryonic content of GA_4_ during incubation of RedlandB2 and IS9530 immature seeds at 20 ºC.

Figure S2. Complete sequences of probes used in EMSA experiments.

Figure S3. Phylogenetic relationships of GA2oxs.

Figure S4. *GA2ox3* probe sequence alignment for *S. bicolor* genotypes IS9530 and RedlandB2.

Figure S5. Domain architecture of group I monocot GA2ox proteins, showing the similarity in domain composition and location.

Figure S6. Pairwise comparison plots between SbGA2ox3 and each M3 promoter (OsGA2ox3, OsGA2ox4, BdGA2ox5, and BdGA2ox8) performed with the EARS tool.

Table S1. GA2ox protein sequence accessions, naming terminology, and database used for eight plant species.

Supplementary Data
