# Supplementary material for: In vitro binding of Sorghum bicolor transcription factors ABI4 and ABI5 to a conserved region of a GA 2-OXIDASE promoter: possible role of this interaction in the expression of seed dormancy
Source: J Exp Bot. 2013 Oct 22;64(18):5721–35. doi: 10.1093/jxb/ert347 (PMC3871824; doi:10.1093/jxb/ert347)
Supplement: Supplementary Data [file supp_64_18_5721__index.html]

In vitro binding of Sorghum bicolor transcription factors ABI4 and ABI5 to a conserved region of a GA 2-OXIDASE promoter: possible role of this interaction in the expression of seed dormancy — In vitro binding of Sorghum bicolor transcription factors ABI4 and ABI5 to a conserved region of a GA 2-OXIDASE promoter: possible role of this interaction in the expression of seed dormancy — Supplementary Data 

# *In vitro* binding of *Sorghum bicolor* transcription factors ABI4 and ABI5 to a conserved region of a *GA 2-OXIDASE* promoter: possible role of this interaction in the expression of seed dormancy

## Supplementary Data

Data files

**Files in this Data Supplement:**

- Supplementary Data - Supplementary Data
