# Supplementary material for: In vitro binding of Sorghum bicolor transcription factors ABI4 and ABI5 to a conserved region of a GA 2-OXIDASE promoter: possible role of this interaction in the expression of seed dormancy
Source: J Exp Bot. 2013 Oct 22;64(18):5721–35. doi: 10.1093/jxb/ert347 (PMC3871824; doi:10.1093/jxb/ert347)
Supplement: Supplementary Data [file supp_ert347_jexbot103655_file001.pdf]

Supplementary Data

In vitro binding of *Sorghum bicolor* transcription factors ABI4 and ABI5 to a conserved region of a *GA 2-OXIDASE* promoter: possible role of this interaction in the expression of seed dormancy

Renata Cantoro; Carlos Daniel Crocco; Roberto Luis Benech-Arnold and María Verónica Rodríguez

Figure S1

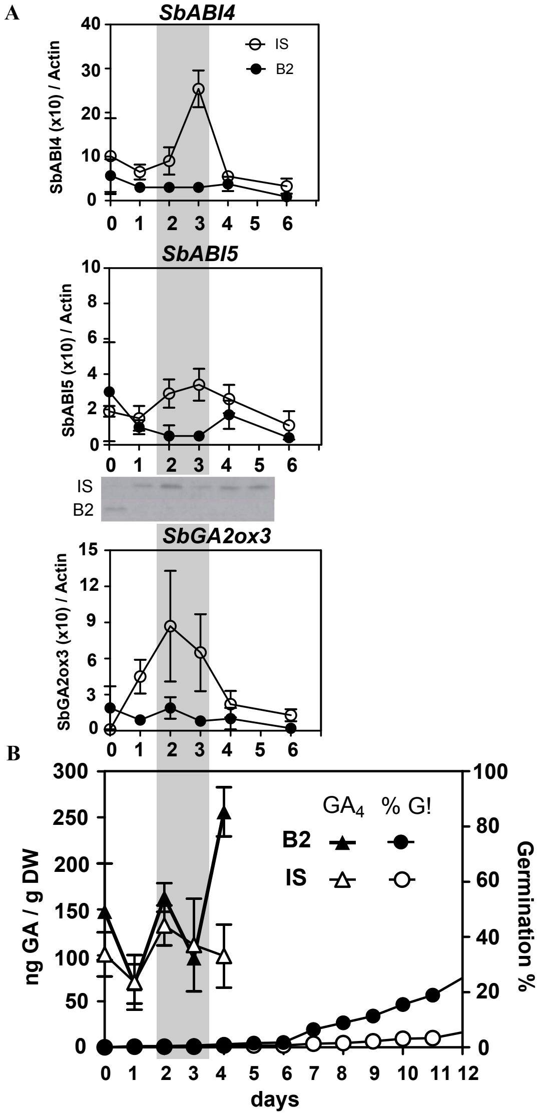

Adapted from Rodríguez *et al*., 2012; 2009.

Figure S1. A, Embryonic transcript levels for *SbABI4, SbABI5* and *SbGA2ox3* genes during 20ºC incubation of immature grains for RedlandB2 (sprouting susceptible, full symbols) and IS9530 (sprouting resistant, empty symbols). Bars indicate the SEM. SbABI5 protein accumulation (detected by western blot) during grain incubation, are shown below *SbABI5* transcript levels for both lines. B, Evolution of germination percentage (circles) and embryonic content of GA_4 (triangles) during incubation time for RedlandB2 (full symbols) and IS9530 (empty symbols) immature seeds, incubated at 20ºC. They grey box indicates temporal coincidence for all components. Results taken from Rodríguez *et al*. (2012; 2009).

Figure S2

***SbGA2ox3* probe (242 bp)**

GGGCGCCGTGGGAAAACTGCTATTGGCCGGCGTG
CCATCGCCTCGTATCGTATGCGTCTCCCATGGAGC
CATGCATGCAGCGCGGCGGGCCGACCTGCCACCG
GTGGATCGCGTCCGGACACGTCGCGCCCCGGACC
CCGACTGCCCTGGCTCCATCGGCACCGTGCACCC
AAAGCGATCCCACCCCCGATGCATCCTTTTCGTCT
CCCTGTCAGCTGGGTCCCATCCAGCCAGGTGCCG
CC

***SbGAMyb* probe (183 bp)**

TCTGGGAAGAACCAACAACCATCGGTCCGAAGTT
CAAGTTCTTCTGCTGGCACACCTTGTGAGACTAC
TACGGTGGTTAGCCCAGAGTTTGATATGGGCCAG
GAATATTGGGAAGAACAGCCTGGTTCTTTCCTCA
GTGAATATGCTCATTTTAGTGGAAATTCTTTGACC
GAATCCACTCCT

***AtEm6* promoter probe (186 bp)**

AGTTAAAGAACACGCGGCGAAGAAGTACGGCCA
CTACCACGTTCAAAGTATACGTCAGCTAATAAAG
TCAGACACGTGGCATGTCACCAAGAAAGAAGAG
CATACGTATGACGTACCGATTGTCCCTGAGTCAC
CACGTCGAACATCCACGATGCAACACAACCAAA
ACGCCCTCCGGATTGA

***SbGA2ox3* probe a (131 bp; intact ABRE and CE)**

GGGCGCCGTGGGAAAACTGCTATTGGCCGGCGTG
CCATCGCCTCGTATCGTATGCGTCTCCCATGGAGC
CATGCATGCAGCGCGGCGGGCCGACCTGCCACCG
GTGGATCGCGTCCGGACACGTCGCGCCC

***SbGA2ox3* probe b (131 bp; mutated ABRE)**

GGGCGCCGTGGGAAAACTGCTATTGGCCGGCGTG
CCATCGCCTCGTATCGTATGCGTCTCCCATGGAGC
CATGCATGCAGCGCGGCGGGCCGACCTGCCACCG
GTGGATCGCGTCCGGACAATTCGCGCCC

***SbGA2ox3* probe c (114 bp; mutated CE and no ABRE included)**

GGGCGCCGTGGGAAAACTGCTATTGGCCGGCGTG
CCATCGCCTCGTATCGTATGCGTCTCCCATGGAGC
CATGCATGCAGCGCGGCGGGCCGACCTGCTTCCG
GTGGATCGCGT

Figure S2. Complete sequences of probes used in EMSA experiments. Mutated bases are highlighted in red in probes b and c.

Figure S3

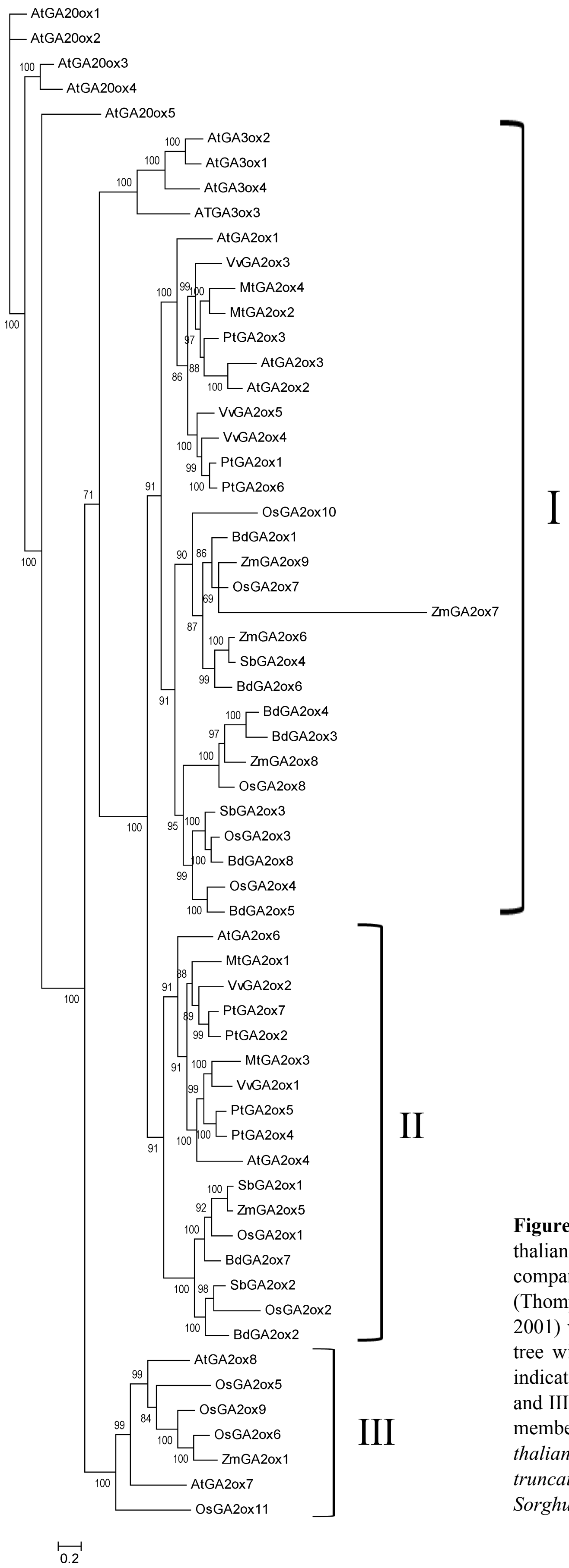

Figure S3. Phylogenetic relationships for GA2oxs. Arabidopsis thaliana GA3oxs and GA2oxs were included for outgroup comparison. Full-length proteins were aligned using ClustalW (Thompson *et al*., 1997). MrBayes (Huelsenbeck & Ronquist, 2001) was used to construct a Bayesian inference phylogenetic tree with 1,500,000 generations. Bootstrap support values are indicated. GA2oxs were divided into three different groups: I, II and III. Members of I and II groups catabolize C19-GAs, while members of III group catabolize C20-GAs. At: *Arabidopsis thaliana*; Bd: *Brachypodium distachyon*; Mt: *Medicago truncatula*; Os: *Oryza sativa*; Pt: *Populus trichocarpa*; Sb: *Sorghum bicolor*; Vv: *Vitis vinifera*; Zm: *Zea mayz.*

Figure S4

```
IS9530     -505   GGGCGCCGTGGGAAAACTGCTATTGGCCGGCGTGCCATCGCCTCGTATCGTATGCGTCTC
RedlandB2  -505   GGGCGCCGTGGGAAAACTGCTATTGGCCGGCGTGCCATCGCCTCGTATCGTATGCGTCTC
                  ************************************************************

IS 9530           CCATGGAGCCATGCATGCAGCGCGGCGGGCCGACCTGCCACCGGTGGATCGCGTCCGGAC
RedlandB2         CCATGGAGCCATGCATGCAGCGCGGCGGGCCGACCTGCCACCGGTGGATCGCGTCCGGAC
                  ************************************************************

IS9530            ACGTCGCGCCCCGGACCCCGACTGCCCTGGCTCCATCGGCACCGTGCACCCAAAGCGATC
RedlandB2         ACGTCGCGCCCCGGACCCCGACTGCCCTGGCTCCATCGGCACCGTGCACCCAAAGCGATC
                  ************************************************************

IS9530            CCACCCCCGATGCATCCTTTTCGTCTCCCTGTCAGCTGGGTCCCATCCAGCCAGGTGCCG
RedlandB2         CCACCCCCGATGCATCCTTTTCGTCTCCCTGCCAGCTGGGTCCCATCCAGCCAGGTGCCG
                  ******************************* ****************************

IS9530            CC  -263
RedlandB2         CC  -263
                  **
```

Figure S4. *GA2ox3* probe sequence alignment for *S. bicolor* genotypes IS9530 and RedlandB2. Sequences were completely aligned with Clustal W software (Thompson *et a*l., 1997).

Figure S5

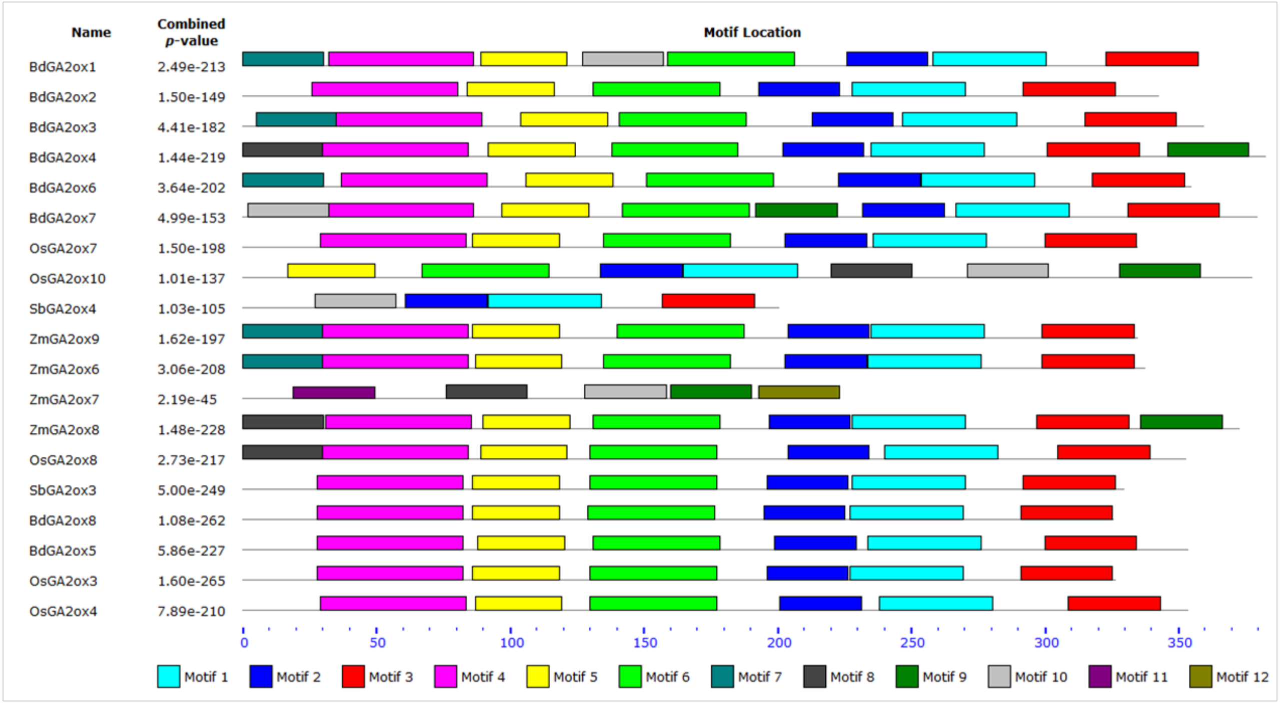

Figure S5. Domain architecture of group I monocot GA2oxs proteins, showing the similarity in domain composition and location. SbGA2ox4 appears as a truncated version of SbGA2ox3.

Figure S6

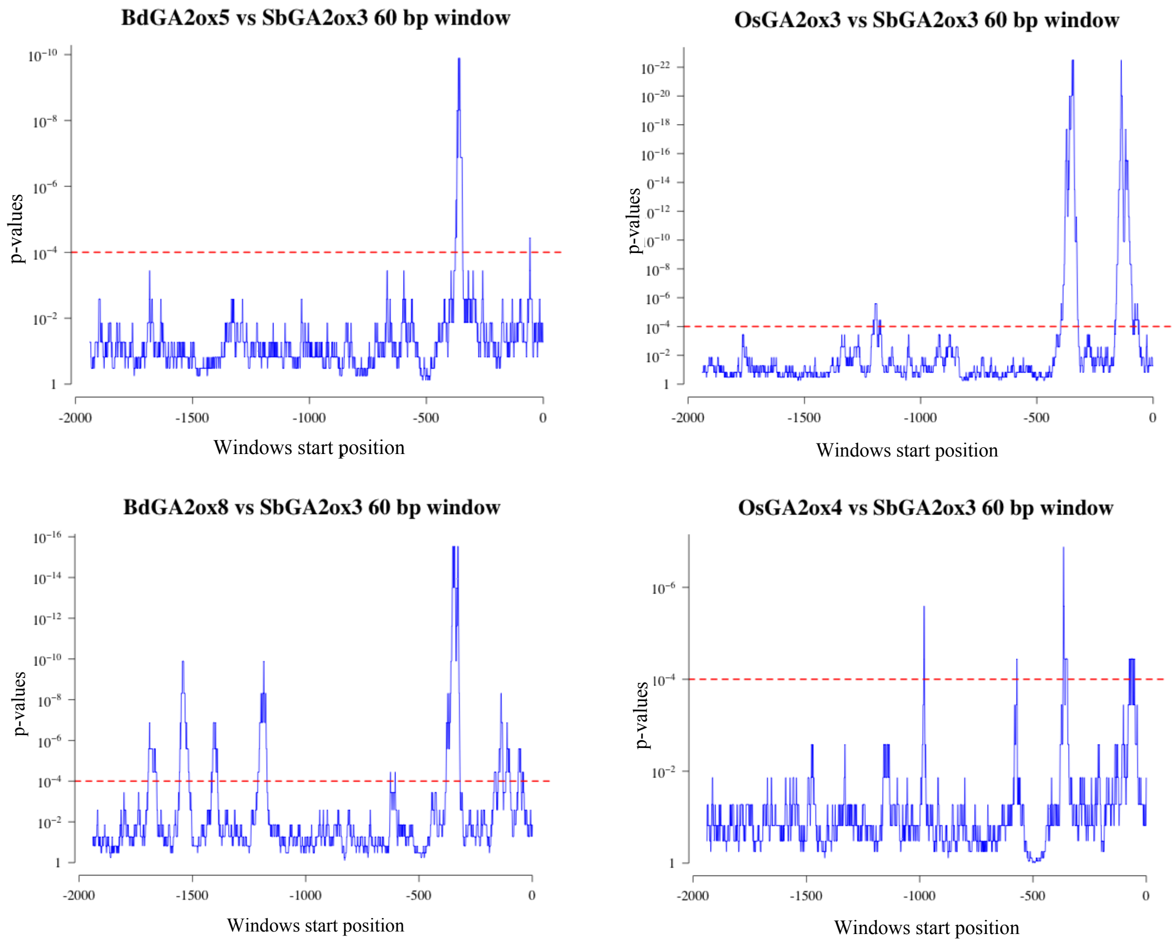

Figure S6. Pair wise comparison plots between SbGA2ox3 and each M3 member promoter (OsGA2ox3, OsGA2ox4, BdGA2ox5 and BdGA2ox8) performed with EARS tool. Red dashed line indicates the selected cut off p-value (0.0001), suggesting the existence of significant peaks 1 and 2 in all the comparisons.

Table S1. GA2ox proteins sequence accessions, naming terminology and data base used for eight plant species.

Specie	ID code	GA2ox name	Database
Arabidopsis thaliana	ath:AT1G78440	GA2ox1	KEGG
	ath:AT1G30040	GA2ox2	
	ath:AT2G34555	GA2ox3	
	ath: AT1G47990	GA2ox4	
	ath: AT1G02400	GA2ox6	
	ath: AT1G50960	GA2ox7	
	ath:AT4G21200	GA2ox8	
	ath:AT1G15550	GA3ox1	
	ath:AT1G80340	GA3ox2	
	ath:AT4G21690	GA3ox3	
	ath:AT1G80330	GA3ox4	
	ath:AT4G25420	GA20ox1	
	ath:AT5G51810	GA20ox2	
	ath:AT5G07200	GA20ox3	
	ath:AT1G60980	GA20ox4	
	ath:AT1G44090	GA20ox5	
Brachypodium distachyon	bdi:100826640	GA2ox1	KEGG
	bdi:100841118	GA2ox2	
	bdi:100835624	GA2ox3	
	bdi:100836239	GA2ox4	
	bdi:100831936	GA2ox5	
	bdi:100821200	GA2ox6	
	bdi:100842224	GA2ox7	
	bdi:100845998	GA2ox8	
Medicago truncatula	MTR_1g086550	GA2ox1	KEGG
	MTR_2g019370	GA2ox2	
	MTR_2g033270	GA2ox3	
	MTR_4g096840	GA2ox4	
Oryza sativa	LOC_Os05g06670	GA2ox1	Rice Genome
	LOC_Os01g22920.1	GA2ox2	
	LOC_Os01g55240.1	GA2ox3	
	LOC_Os05g43880.1	GA2ox4	
	LOC_Os07g01340.1	GA2ox5	
	LOC_Os04g44150.1	GA2ox6	
	LOC_Os01g11150.1	GA2ox7	
	LOC_Os05g48700.1	GA2ox8	
	LOC_Os02g41954.1	GA2ox9	
	LOC_Os05g11810.1	GA2ox10	
	LOC_Os04g33360.1	GA2ox11	
Populus trichocarpa	POPTR_550316	GA2ox1	KEGG
	POPTR_409194	GA2ox2	
	POPTR_758699	GA2ox3	
	POPTR_765885	GA2ox4	
	POPTR_723959	GA2ox5	

	POPTR_822902<br>POPTR_245496	GA2ox6<br>GA2ox7	
Sorghum bicolor	Sb09g004520<br>Sb03g013450<br>Sb03g035000<br>Sb09g006490	GA2ox1<br>GA2ox2<br>GA2ox3<br>GA2ox4	KEGG
Vitis vinifera	vvi:100258599<br>vvi:100248676<br>vvi: 100244543<br>vvi:100264177<br>vvi:100248356	GA2ox1<br>GA2ox2<br>GA2ox3<br>GA2ox4<br>GA2ox5	KEGG
Zea mayz	zma:100281860<br>zma:100285694<br>zma:100280480<br>zma:100272456<br>zma:100192514<br>zma:100284370	GA2ox1<br>GA2ox5<br>GA2ox6<br>GA2ox7<br>GA2ox8<br>GA2ox9	KEGG
